# Temporal variability in trace metal solubility in a paddy soil not reflected in uptake by rice (*Oryza sativa* L.)

**DOI:** 10.1007/s10653-016-9803-7

**Published:** 2016-01-30

**Authors:** Yunyu Pan, Gerwin F. Koopmans, Luc T. C. Bonten, Jing Song, Yongming Luo, Erwin J. M. Temminghoff, Rob N. J. Comans

**Affiliations:** 1 Key Laboratory of Soil Environment and Pollution Remediation, Institute of Soil Science, Chinese Academy of Sciences, Nanjing, 210008 People’s Republic of China; 2 Department of Soil Quality, Wageningen University, P.O. Box 47, 6700 AA Wageningen, The Netherlands; 3 Alterra, P.O. Box 47, 6700 AA Wageningen, The Netherlands; 4 Yantai Institute of Costal Zone Research, Chinese Academy of Sciences, Yantai, 264003 People’s Republic of China

**Keywords:** Paddy soils, Trace metal contamination, Water management, Redox potential, Bioavailability, Uptake

## Abstract

Alternating flooding and drainage conditions have a strong influence on redox chemistry and the solubility of trace metals in paddy soils. However, current knowledge of how the effects of water management on trace metal solubility are linked to trace metal uptake by rice plants over time is still limited. Here, a field-contaminated paddy soil was subjected to two flooding and drainage cycles in a pot experiment with two rice plant cultivars, exhibiting either high or low Cd accumulation characteristics. Flooding led to a strong vertical gradient in the redox potential (Eh). The pH and Mn, Fe, and dissolved organic carbon concentrations increased with decreasing Eh and vice versa. During flooding, trace metal solubility decreased markedly, probably due to sulfide mineral precipitation. Despite its low solubility, the Cd content in rice grains exceeded the food quality standards for both cultivars. Trace metal contents in different rice plant tissues (roots, stem, and leaves) increased at a constant rate during the first flooding and drainage cycle but decreased after reaching a maximum during the second cycle. As such, the high temporal variability in trace metal solubility was not reflected in trace metal uptake by rice plants over time. This might be due to the presence of aerobic conditions and a consequent higher trace metal solubility near the root surface, even during flooding. Trace metal solubility in the rhizosphere should be considered when linking water management to trace metal uptake by rice over time.

## Introduction

Contamination of paddy soils with trace metals due to mining and smelting activities, application of fertilizers and sewage sludge, and wastewater irrigation is a widespread problem (Kuo et al. 2006; Römkens et al. 2009a; Zeng et al. 2011). Trace metal contamination of paddy soils can lead to the accumulation of trace metals in rice grains (Römkens et al. 2009b; Zeng et al. 2011). Since rice is one of the most important staple foods for human beings in South and Southeast Asia (Römkens et al. 2009a), consumption of rice with elevated trace metal levels is a serious threat to food safety and human health (Römkens et al. 2009b; Meharg et al. 2013). For typical rice-eating populations, the dietary intake of Cd via rice consumption is the major exposure pathway for human beings to Cd (Tsukahara et al. 2003; Meharg et al. 2013). For minimizing human health risks, it is important to understand the processes controlling the uptake of trace metals by rice plants.

The uptake of trace metals by rice plants depends on the bioavailability of trace metals in paddy soils (Simmons et al. 2008; Römkens et al. 2009b). For trace metals in paddy soils, three pools with a different bioavailability can be distinguished: the total, reactive, and directly available trace metal pools (Römkens et al. 2009a). The reactive pool represents trace metals adsorbed to reactive surfaces of soil organic matter (SOM), short-range ordered metal-(hydr)oxides, and clay, and it controls the trace metal concentrations in soil solution (Weng et al. 2001; Tipping et al. 2003). The size of this pool can be determined by an extraction of soil with 0.05 M EDTA, 0.1 M HCl, or 0.43 M HNO_3_ (Römkens et al. 2009a). The directly available pool represents the free or total dissolved trace metal concentration in soil solution which can be directly related to the uptake of trace metals by plants (Lofts et al. 2004; Peijnenburg et al. 2007). The size of this pool can be determined by either sampling of the soil solution with soil moisture samplers or lysimeters (Reynolds et al. 2004; Shen and Hoffland 2007) or it can be mimicked by an extraction of soil with weak salt extracts such as CaCl_2_, Ca(NO_3_)_2_, or NaNO_3_ (Houba et al. 2000; Peijnenburg et al. 2007). The size of the total pool is normally larger than the size of the reactive pool, and the difference between both pools is interpreted to be nonreactive on a time scale relevant to the duration of a cropping season for rice (Römkens et al. 2009a). For paddy soils varying widely in soil properties such as pH and cation exchange capacity (CEC), regression-based log–log relationships have been used to link the directly available Cd pool as determined by an 0.01 M CaCl_2_ extraction of soil to the Cd content of rice grains (Simmons et al. 2008; Römkens et al. 2009b). Other studies linked the Cd content in rice grains to the reactive Cd pool (0.43 M HNO_3_) in combination with soil properties including pH, SOM, and clay (Brus et al. 2009) or pH and CEC (Römkens et al. 2009b). These soil properties were included because they affect the solid–solution partitioning of trace metals (Weng et al. 2001). However, most of the relationships in the aforementioned studies were determined for ripened and harvested rice grains.

Rice is usually cropped in lowland paddy fields. Flooding conditions prevail during almost the entire cropping period, because lowland rice is extremely sensitive to water shortage (Kögel-Knabner et al. 2010). Flooded paddy fields are usually drained during the late tillering stage to control ineffective tillering of the rice plants and shortly before harvest to enable faster rice grain ripening and to facilitate harvesting. Consequently, lowland paddy fields undergo at least two flooding and draining cycles, which are known to lead to fluctuations in the redox conditions (Kögel-Knabner et al. 2010; Pan et al. 2014). Upon flooding, the redox potential (Eh) will decrease and the reduction of Mn- and Fe-(hydr)oxides will lead to their dissolution while organic matter will be released, resulting in elevated levels of Mn^2+^ and Fe^2+^ and dissolved organic matter (DOM) in soil solution (Grybos et al. 2007; Pan et al. 2014). Since SOM and metal-(hydr)oxides play an important role in the adsorption of trace metals in soils (Weng et al. 2001), a decrease in the reactive surfaces of these soil constituents can lead to an increase in the solubility of trace metals. The mobilization of trace metals might be even further amplified by the release of DOM after flooding (Pan et al. 2014), because trace metals including Cu and Pb can bind strongly to DOM (Amery et al. 2007; Koopmans and Groenenberg 2011). On the other hand, reduction of SO_4_^2−^ to S^2−^ can cause trace metals to precipitate as poorly soluble minerals (de Livera et al. 2011; Fulda et al. 2013). Additionally, the pH of acidic soils can increase during flooding (Kögel-Knabner et al. 2010), which enhances the adsorption of trace metals by SOM and metal-(hydr)oxides (Weng et al. 2001). During drainage and subsequent oxidation of paddy soils, the above-mentioned reduction reactions can reverse, leading either to the retention of trace metals via adsorption to freshly formed Mn (III/IV)- and Fe(III)-(hydr)oxides or to their release via dissolution of trace metal sulfide precipitates. Also, the pH can return to its antecedent value, thereby influencing the adsorption of trace metals by SOM and metal-(hydr)oxides. These complex and dynamic effects of flooding and drainage on the Eh and redox chemistry of paddy soils can lead to a strong temporal variability in trace metal solubility (Du Laing et al. 2009; Schulz-Zunkel and Krueger 2009; Shaheen et al. 2014), which, in turn, might affect trace metal uptake by rice plants. However, current knowledge of how the effects of water management of paddy fields on trace metal solubility are linked to uptake of trace metals by rice plants over time is still limited.

In our previous work, we investigated how alternating flooding and draining conditions affected redox chemistry and solubility of heavy metals in two field-contaminated soils with similar properties but a contrasting pH (Pan et al. 2014, 2016). The primary objective of our current study is to investigate the relationship between the solubility of trace metals and their uptake by rice plants over time. To realize this objective, we carefully simulated two consecutive flooding and drainage cycles during rice growth using an acidic field-contaminated paddy soil in a pot experiment. Besides trace metal bioavailability in paddy soils, rice plant-specific factors can have a profound influence on the accumulation of trace metals in rice grains (Yu et al. 2006; Römkens et al. 2009b). For example, Indica and Japonica rice plant cultivars have been demonstrated to differ substantially in their ability to translocate Cd from the roots into their shoots, with a higher ratio of Cd in rice grains to Cd in the roots for Indica than for Japonica (Römkens et al. 2009b). Additionally, considerable variation in Cd accumulation in rice grains has been identified among various Indica varieties (Römkens et al. 2009b; Hu et al. 2013). Therefore, the secondary objective of our study was to quantify the differences in Cd content in rice grains of two Indica rice plant cultivars with different Cd accumulation characteristics. The Eh was monitored continuously at five depths along the soil profile during the two successive flooding and drainage cycles in the pot experiment. Soil solution was sampled using soil moisture samplers during the major rice growth stages at the same depths to determine its chemical composition including pH and the concentrations of total dissolved Mn, Fe, and trace metals and dissolved organic carbon (DOC). The trace metal contents in different rice plant tissues including the roots, stem, leaves, husks, and brown rice were measured during the major rice growth stages. This experimental approach allowed us to link the solubility of trace metals to their uptake by rice plants over time.

## Materials and methods

### Soil collection

Soil with a clay loam texture was sampled from a paddy field (0–20 cm) nearby a former transformer and electronic waste stripping and recycling factory in Taizhou city, Zhejiang Province, P.R. China (Pan et al. 2014). This soil was severely contaminated with trace metals because of industrial activities in the proximity of the sampling site (Sun et al. 2013). The soil was air-dried, passed through a 2-mm sieve, and stored at room temperature before analyzing the soil characteristics and preparing the pot experiment.

### Experimental setup of the pot experiment

**Fig. 1:**
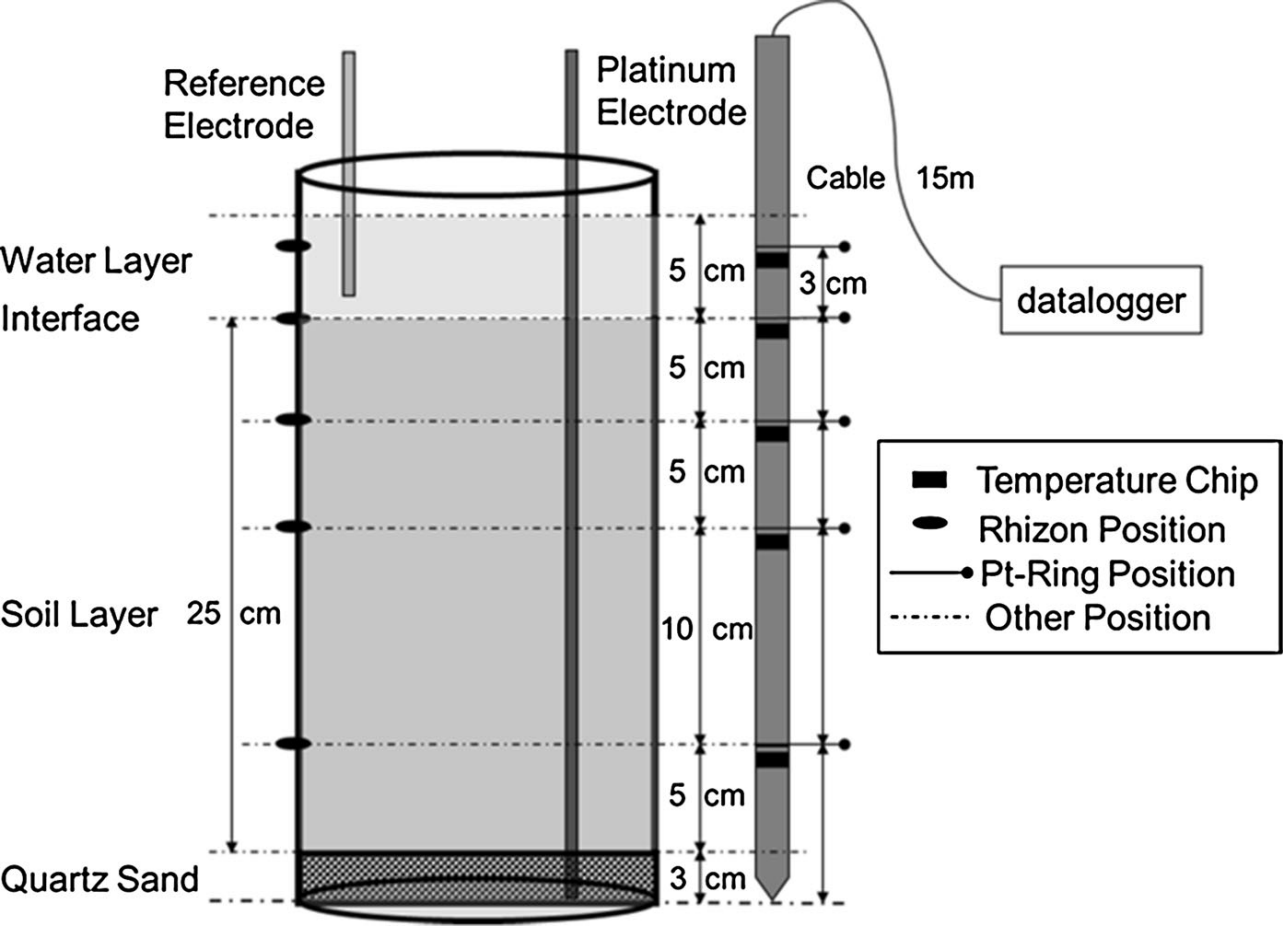
Experimental setup of the pots used to monitor the Eh and chemical composition of the soil solution in the pot experiment with the two rice plant cultivars

**Fig. 2:**
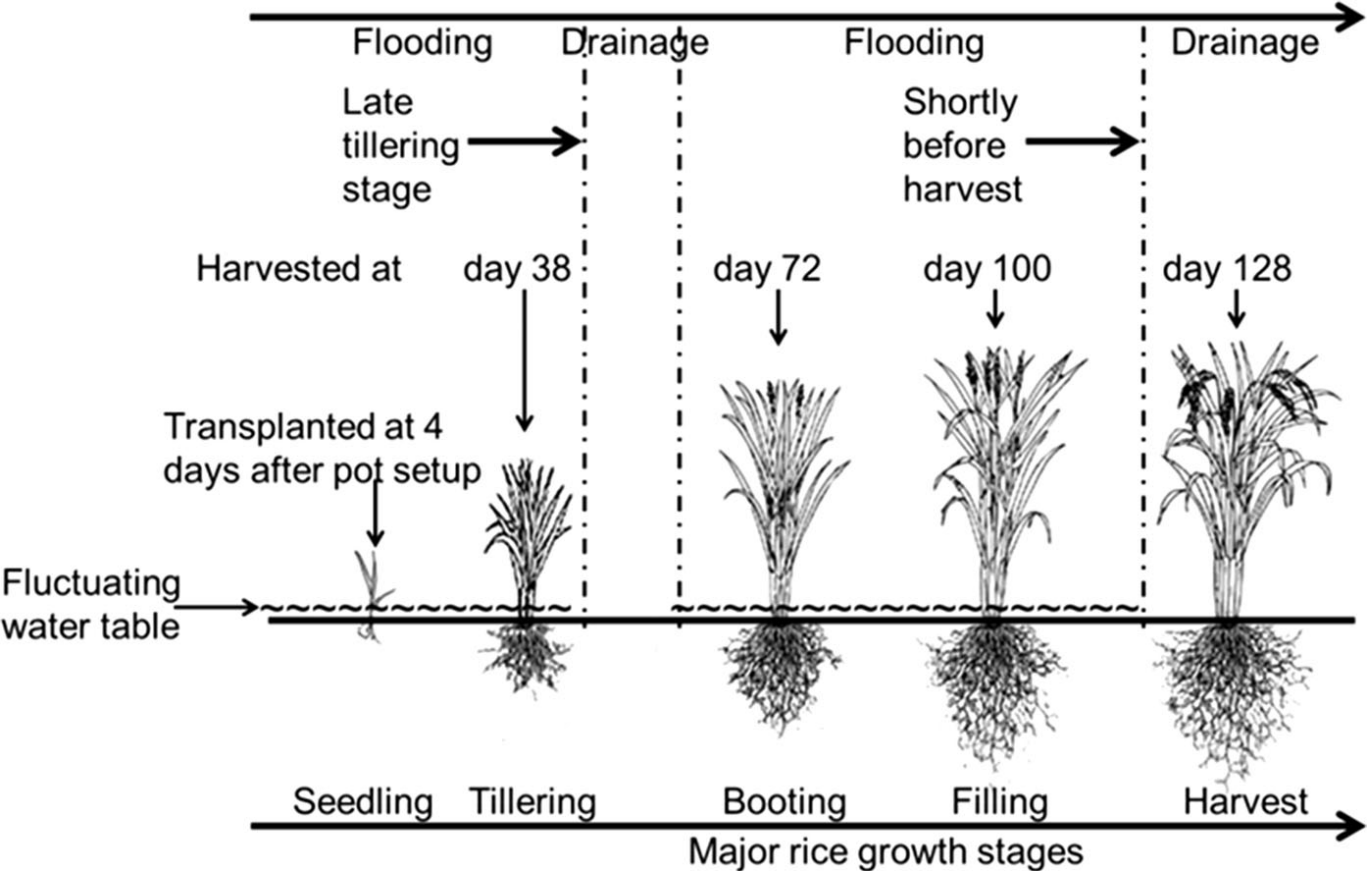
Harvesting times of the rice plants during the major rice growth stages and alternating flooding and drainage periods in the pot experiment. The first drainage period of 8 days was after 58 days of flooding, and the second drainage period of 16 days was after 46 days of flooding

For the pot experiment, pots made of PVC material with a height of 35 cm and a diameter of 18 cm were used (Fig. 1). Quartz sand, pretreated and cleaned with 0.14 M HNO_3_ and deionized water, was placed at the bottom of the pot in a layer of 3 cm (Fig. 1). A piece of 1-mm nylon sieve material was put on top of the quartz sand. Each pot was filled with 4.5 kg of air-dried paddy soil. Before transferring the soil to each pot, it was thoroughly mixed with basal N, P, and K fertilizer including 0.83 g N as urea (CO[NH_2_]_2_), 0.23 g P as KH_2_PO_4_, and 0.52 g K as KCl. During mixing, the soil moisture content was adjusted to about 30 % (w:w) to facilitate water penetration in the potted soil during the first flooding period. The thickness of the potted soil layer was 25 cm. In the pot experiment, two different rice cultivars were used (see “Management and harvesting of the rice plants” section). The pot experiment was set up with 12 replicates for each of the two rice cultivars (24 pots in total) in a greenhouse. The temperature and light intensity in the greenhouse were regulated in accordance with the ambient weather conditions, because an optimal rice growth requires a high light intensity and temperature. The pot experiment was subjected to two successive flooding and drainage cycles, which is in accordance with the water management of lowland paddy rice fields in practice: one drainage period at the late tillering growth stage and one drainage period shortly before the harvest (Fig. 2). The first flooding period lasted for 58 days until the late tillering stage and was followed by a drainage period of 8 days aiming to control ineffective tillering. The second flooding period lasted for 46 days until the ripening stage and was followed by a drainage period of 16 days to enable faster rice ripening and to facilitate harvesting. For flooding of the pots, three liter of deionized water was added to each pot in a step-wise manner leaving a final water layer on top of the soil layer of 5 cm (Fig. 1). The flooding procedure was completed within 3 h for all 24 pots. For each rice plant cultivar, three pots were randomly chosen to monitor the Eh and chemical composition of the soil solution. For Eh monitoring, a combined Eh-temperature glass fiber probe (Paleoterra Products, Amsterdam, The Netherlands) was inserted vertically in each of the six pots. The probes were tailor-made with five platinum Eh measurement points enabling us to monitor the Eh 3 cm above the soil–water interface (+3 cm), at the interface (0 cm), and at depths of 5 (−5 cm), 10 (−10 cm), and 20 cm (−20 cm) below the interface (Fig. 1). The Ag–AgCl reference electrodes were positioned in the water layer. The Eh signal was automatically logged every 15 min. The Eh relative to the standard hydrogen electrode was calculated from the measured potential with a temperature-dependent correction for the potential of the Ag–AgCl reference electrode. The temperature was measured using the combined Eh-temperature probe by platinum electrodes at five positions within the pots: +3, 0, −5, −10, and −20 cm (Fig. 1). For collecting soil solution samples from the pots, five soil moisture samplers (SMS) (Rhizosphere Research Products, Wageningen, The Netherlands) with a diameter of 2.5 mm, a mean pore size of 0.15 µm, and a porous section of 10 cm were placed horizontally at the same depths (+3, 0, −5, −10, and −20 cm) in the pot at which the Eh was measured. The silicone connector of the SMS tightly sealed off the holes of the pots, preventing air from penetrating the pots. Soil solution samples were taken with a vacuumed syringe at 11 occasions during the major rice growth stages (Fig. 2). This syringe was connected to the female luer lock of the SMS, which was closed again immediately after sampling. The soil solution samples were split in two subsamples: In one subsample, metal and sulfur concentrations were measured after acidification by adding concentrated HNO_3_ to a level of 1.2 % (v:v), whereas the other subsample was used for the measurement of pH and DOC. The samples were stored in polypropylene tubes at 4 °C until chemical analysis as further detailed in the “Chemical analysis” section.

### Management and harvesting of the rice plants

Two Indica varieties were used in our pot experiment: Zhongxiang 1 (A16) and Indonesia (A159). These cultivars are commonly grown in Zhejiang Province, P.R. China. Under conventional water management conditions, cultivar A16 has a lower potential to accumulate Cd in brown rice than cultivar A159 (Hu et al. 2013). The seeds of both cultivars were provided by the China National Rice Research Institute (Hangzhou City, Zhejiang Province, P.R. China) and disinfected with 10 % H_2_O_2_ solution (v:v) before use. After germination, germinated seeds were grown in an uncontaminated soil for 30 days before transplanting the seedlings to the pots. Four days after setting up the pots and flooding the potted soil for the first time, three clusters, with each cluster containing 6 seedlings, were transplanted to each pot. The clusters were evenly positioned over the soil surface of each pot. During rice growth, an extra amount of 0.35 g N as urea was applied to each pot at the early rice tillering stage (day 10). An extra similar dose of urea was applied at the early rice grain filling stage (day 80). A water layer of 5 cm above the soil surface of each pot was maintained by adding deionized water three times each day at 7 a.m., 2 p.m., and 7 p.m. Three pots for each cultivar were destructively harvested at the rice tillering stage (day 38), booting stage (day 72), filling stage (day 100), and ripening stage (day 128) (Fig. 2). The harvested rice plants were first thoroughly washed with tap water and then with deionized water to remove dust and adhering soil particles. Subsequently, the rice plants were separated into different plant tissues including roots, stem, leaves, and grains. These tissues were wrapped in a clean paper envelope and oven-dried at 105 °C for 30 min and further dried at 80 °C to a constant weight. The dried grains were then further separated into the husks and brown rice. Dry weight was determined for all rice tissues. Subsequently, all rice tissues were milled by a stainless steel grinding machine to measure the trace metal contents (see “Chemical analysis” section).

### Chemical analysis

#### Soil analyses

The pH was measured in a settling 1:10 (w:v) suspension of soil in 0.01 M CaCl_2_ (Houba et al. 2000); the CEC was measured using 1 M NH_4_OAc buffered at pH 7.0 (USDA 1996), SOM by loss-on-ignition (550 °C) and clay by the sieve and pipette method (Houba et al. 1997). Dissolved organic carbon and sulfur were measured in the same CaCl_2_ extract as used for the measurement of pH after filtration through a 0.45-μm filter (Schleicher & Schuell 602H). Dissolved organic carbon was measured by a TOC analyzer (Multi C/N 3000). Sulfur was measured by inductively coupled plasma atomic emission spectrometry (ICP-AES; IRIS Intrepid II) and ion chromatography (Dionex, ICS-2100) (Dick and Tabatabai 1979). Ion chromatography specifically measures the inorganic SO_4_^2−^ form of sulfur. Since the results of both ICP-AES and ion chromatography were very similar (results not shown), we, therefore, interpreted sulfur measured in the CaCl_2_ extract by ICP-AES as S–SO_4_^2−^ (Pan et al. 2014). Short-range ordered Al-, Fe-, and Mn-(hydr)oxides were determined by using the acid ammonium oxalate extraction method (Novozamsky et al. 1986). The Al, Fe, and Mn concentrations in the ammonium oxalate extracts (i.e., Al-ox, Fe-ox, and Mn-ox) were measured by ICP-AES. The size of the reactive trace metal pools was determined by an extraction of soil with 0.43 M HNO_3,_ whereas the size of the total trace metal pools was determined by soil digestion with Aqua Regia (Houba et al. 1997). The Cd, Cu, Ni, Pb, and Zn concentrations in the 0.43 M HNO_3_ extracts and Aqua Regia digests were measured by ICP-AES. Samples with trace metal concentrations below the detection limit of the ICP-AES were re-measured by an inductively coupled plasma mass spectrometer (ICP-MS; Thermo Electron X7). All physico-chemical soil properties were determined in triplicate, and their average ± standard deviation is presented in Table 1.

**Table 1:** Selected physico-chemical properties of the paddy soil used in the pot experiment

Soil property	Unit	Value	Standard^g^
Clay (<2 µm)^a^	%	19.7 ± 0.1	–
SOM^b^	%	9.8 ± 0.1	–
pH^c^		5.2 ± 0.1	–
DOC^c^	mg C L^−1^	150 ± 5	
S-SO_4_^2−c^	mmol kg^−1^	4.2 ± 0.1	
CEC	cmol [+] kg^−1^	11.5 ± 0.3	–
Al-ox^d^	mmol kg^−1^	38 ± 1	–
Fe-ox^d^	mmol kg^−1^	95 ± 1	–
Mn-ox^d^	mmol kg^−1^	1.7 ± 0.1	–
Reactive Cu^e^	mmol kg^−1^	3.97 ± 0.03	–
Total Cu^f^	mmol kg^−1^	7.38 ± 0.08	0.79
Reactive Cd^e^	μmol kg^−1^	50.1 ± 0.2	–
Total Cd^f^	μmol kg^−1^	62.0 ± 0.5	2.7
Reactive Pb^e^	μmol kg^−1^	145 ± 5	–
Total Pb^f^	μmol kg^−1^	222 ± 10	1207
Reactive Zn^e^	mmol kg^−1^	0.60 ± 0.03	–
Total Zn^f^	mmol kg^−1^	2.31 ± 0.08	3.06
Reactive Ni^e^	μmol kg^−1^	164 ± 5	–
Total Ni^f^	μmol kg^−1^	767 ± 17	682

All soil properties were determined in triplicate and their average ± standard deviation is presented

^a^Clay by the sieve and pipette method (Houba et al. 1997)

^b^Soil organic matter (SOM) by loss-on-ignition (550 °C) (Houba et al. 1997)

^c^1:10 (w:v) 0.01 M CaCl_2_ extraction (Houba et al. 2000)

^d^Extraction with acid ammonium oxalate (Novozamsky et al. 1986)

^e^1:10 (w:v) 0.43 M HNO_3_ (Houba et al. 1997)

^f^Aqua Regia (Houba et al. 1997)

^g^Grade II (pH < 6.5) of the Chinese environmental quality standard for soils (GB 15618-1995)

#### Soil solution analyses

The pH of the soil solution samples was measured directly after sampling with a combined glass electrode, and DOC was measured by a TOC analyzer. In the acidified subsamples, Fe, Mn, and S were measured by ICP-AES and Cd, Cu, Ni, Pb, and Zn by ICP-MS.

#### Plant analysis

For analyzing the trace metal contents in the different rice plant tissues, 0.5 g milled tissue material was digested by using a mixture of 5 ml HNO_3_ and 3 ml HClO_4_ (Huang and Schulte 1985). The Cu, Zn, Pb, Cd, and Ni concentrations in the digests were measured by ICP-MS.

## Results and discussion

### Physico-chemical soil properties

Selected physico-chemical properties of the paddy soil are summarized in Table 1. The total Cd, Cu, Ni, and Zn contents exceed the Chinese environmental quality standard for soils (GB 15618-1995; Grade II for soil pH < 6.5) about 23 (Cd), 9 (Cu), and 1.1 (Ni) times, respectively. Hence, this soil is especially heavily contaminated with Cd and Cu. The soil is acidic and the size of the reactive trace metal pools (0.43 M HNO_3_) relative to the total pools (Aqua Regia) increases in the order Ni (21 %) < Zn (26 %) < Cu (54 %) < Pb (65 %) < Cd (81 %). Consequently, Cd is mostly present in highly reactive forms, which can exchange with the soil solution and become available with time for uptake by plants, whereas Zn and Ni are largely contained within chemically inert forms which are rather immobile (Römkens et al. 2009a). These results are very similar to those of Römkens et al. (2009a) for paddy soils with similar physico-chemical properties. The available S–SO_4_^2−^ content in our paddy soil (Table 1) was in the upper range of the available S–SO_4_^2−^ contents (0.06–6.6 mmol kg^−1^) reported for other paddy soils in different regions (Lefroy et al. 1992; Fulda et al. 2013).

### Effect of alternating flooding and drainage on Eh, pH, and Mn, Fe, S, and DOC concentrations

**Fig. 3:**
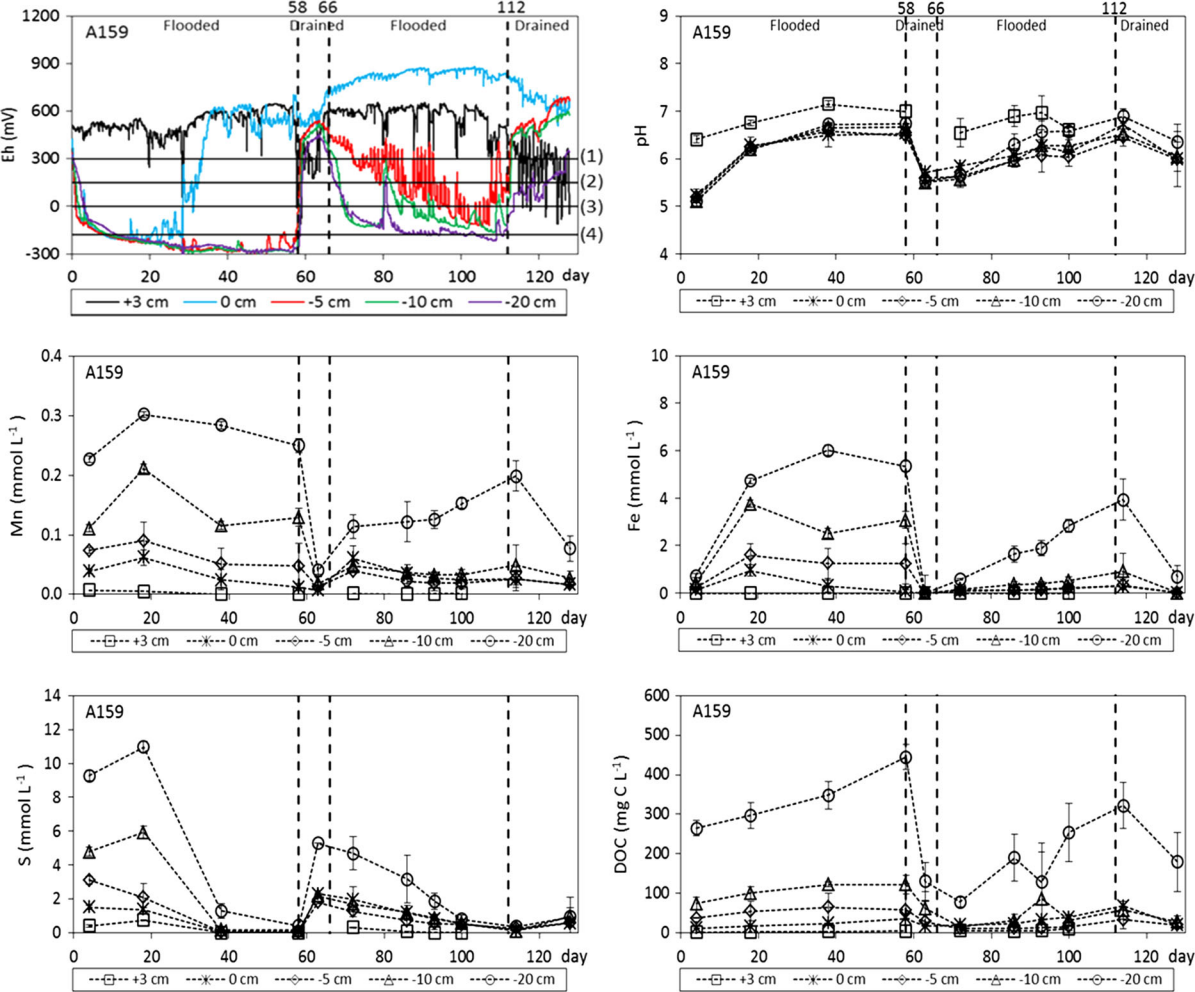
Eh, pH, and Mn, Fe, S, and DOC concentrations in the soil solution for the pot experiment with rice plant cultivar A159 (+3 cm refers to 3 cm above the interface, 0 cm equals the interface between the soil surface and the water layer, and −5, −10 cm, −20 cm refer to 5, 10, and 20 cm below the interface, respectively). *The numbers at the top* *x*-axis of the *two upper figures* indicate the time at which the drainage and flooding periods started. In figure with Eh data, 1–4 refer to the following reactions: (1) denitrification of NO_3_^−^ to N_2_, (2) and (3) reductive dissolution of MnO_2_ and Fe(OH)_3_ to Mn^2+^ and Fe^2+^, respectively, and (4) reduction of SO_4_^2−^ to S^2−^

In Fig. 3, the Eh, pH, and Mn, Fe, S, and DOC concentrations in the overlying water layer and the soil solution of the different soil layers are presented for the pot experiment with rice plant cultivar A159. Since the results for cultivar A16 are very similar to those for cultivar A159, the results for the former cultivar are presented in the Appendix (Fig. 7). The Eh showed a strong contrast between the water layer and the three lower soil layers: Aerobic conditions were maintained in the water layer with an Eh of around 500 mV throughout the pot experiment, whereas anaerobic conditions with an Eh as low as about −300 mV were present at a depth of 5, 10, and 20 cm below the soil–water interface during the first flooding period. The Eh at the interface between the soil surface and the water layer decreased to −200 mV during the first 20 days, then increased to about 550 mV, and remained high during the remainder of the pot experiment. During the first drainage period, the Eh rapidly reached a level of 400 mV in all soil layers, due to re-aeration and oxidation of the potted soil. During the second flooding period, the Eh decreased to a level of about −200 mV in the two lower soil layers. The Eh at a depth of 5 cm was higher than the Eh in the two lower soil layers, but the temporal variability was high. From visual observations during the sampling of rice plant roots, most of the rice plant roots appeared to be present in the upper 5 cm of the potted soil. Therefore, the high temporal variation in Eh at a depth of 5 cm, which had a diurnal pattern, is probably caused by internal aeration of the rice plants during the daily photosynthesis period, leading to downward transport of O_2_ from their shoots to the submerged roots and leakage of O_2_ to the local surrounding soil environment (Colmer 2003; Mongon et al. 2014). Finally, aerobic conditions prevailed in all soil layers during the second drainage period except for the bottom layer, which remained slightly anaerobic.

According to the measured Eh range for our paddy soil, redox reactions including denitrification and reduction of Mn(III/IV) to Mn(II), Fe(III) to Fe(II), and SO_4_^2−^ to S^2−^ can occur (Fig. 3). Hence, reductive dissolution of Mn- and Fe-(hydr)oxides during flooding can explain the clearly elevated Mn and Fe concentrations in soil solution in the three lower soil layers as compared to those in the water layer. The lower Mn and Fe concentrations during drainage and subsequent oxidation of the paddy soil are probably due to precipitation of these metals in the form of Mn (III/IV)- and Fe(III)-(hydr)oxides (Schulz-Zunkel and Krueger 2009; Shaheen et al. 2014). Reduction of SO_4_^2−^ to S^2−^ is likely to occur in the three lower soil layers during flooding according to the Eh values measured in these layers. Therefore, the gradual decrease in the sulfur concentration in soil solution over time can be explained by the formation of poorly soluble sulfide minerals (Murase and Kimura 1997; Du Laing et al. 2009; Schulz-Zunkel and Krueger 2009). Since all above-mentioned reduction reactions consume protons, the pH increased from acidic to an almost neutral level in all soil layers during flooding, whereas the pH in the water layer remained relatively constant. During drainage, the pH in all soil layers decreased again. The DOC concentrations in the three lower anaerobic soil layers during flooding were clearly elevated compared to those in the aerobic overlying water layer. This can be attributed to reductive dissolution of Mn- and Fe-(hydr)oxides and the elevated pH, both leading to the release of adsorbed DOM (Kaiser et al. 1997; Pan et al. 2014). Also, the release of fermentation products might have contributed to the elevated DOC concentrations, because mineralization of organic matter can be incomplete under anaerobic conditions (Vink et al. 2010). The DOC concentrations decreased in all soil layers during drainage.

### Effects of alternating flooding and drainage conditions on the solubility of Cu, Cd, Pb, Zn, and Ni

**Fig. 4:**
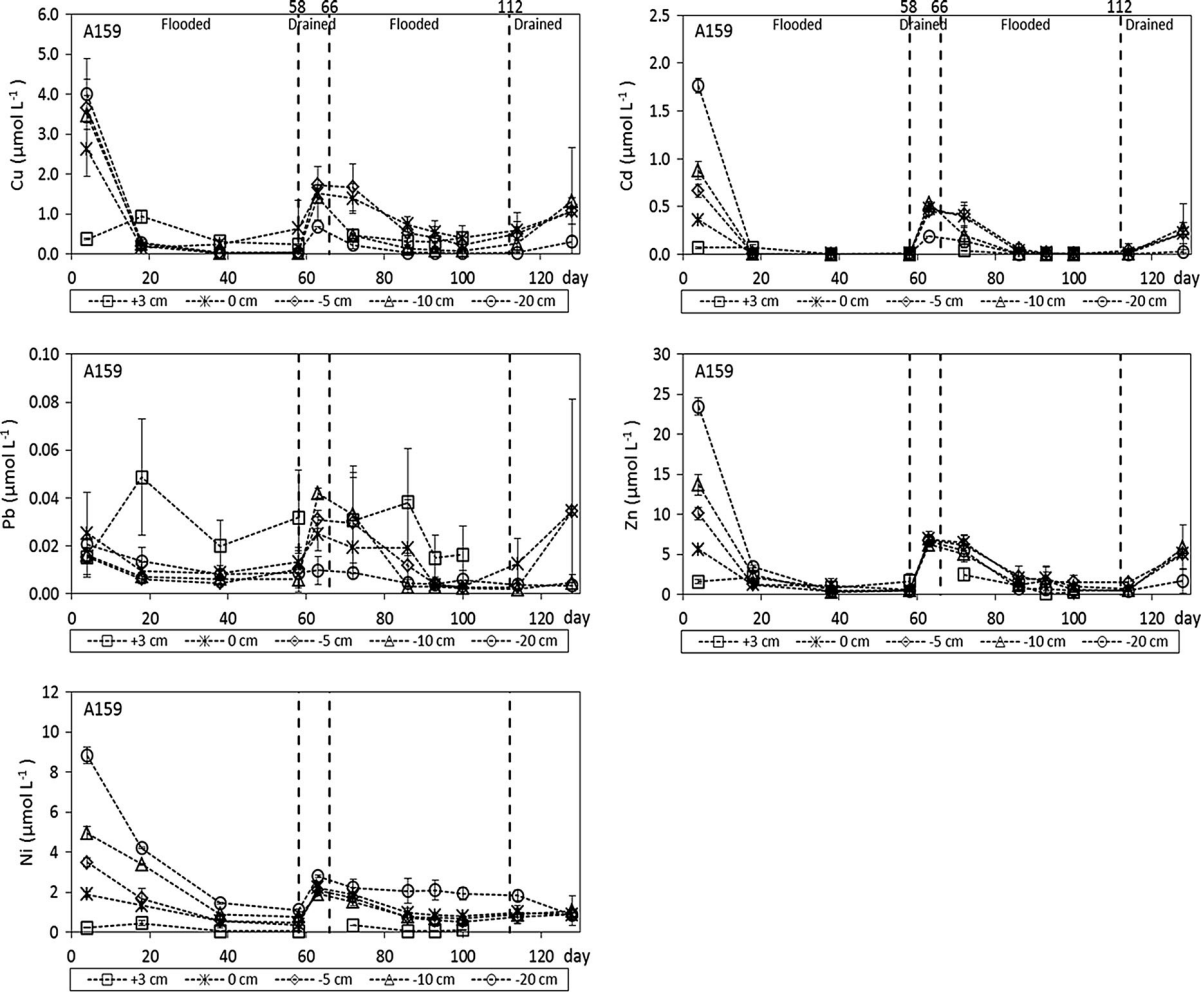
Total dissolved concentrations of Cu, Cd, Pb, Zn, and Ni in the soil solution for the pot experiment with rice plant cultivar A159 (+3 cm refers to 3 cm above the interface, 0 cm equals the interface between the soil surface and the water layer, and −5, −10 cm, −20 cm refer to 5, 10, and 20 cm below the interface, respectively). *The numbers at the top* *x*-axis of the *two upper figures* indicate the time at which the drainage and flooding periods started

In Fig. 4, the trace metal concentrations in the overlying water layer and the soil solution of the different soil layers are presented for the pot experiment with rice plant cultivar A159. The results for cultivar A16 are very similar to those for cultivar A159 and are, therefore, presented in the Appendix (Fig. 8). At the initial sampling occasion (day 4), the solubility of all trace metals except Pb clearly increased with depth. This gradient is probably due to vertical displacement of trace metals and DOC (Fig. 3) via downward transport during the saturation of the potted soil by adding deionized water at the top of the soil surface. In a previous column experiment with the same paddy soil, as described in Pan et al. (2014, 2016), the opposite trend was found for the initial sampling occasion, with trace metal and DOC concentrations clearly decreasing with depth. However, this could be explained by Pan et al. (2014, 2016) by upward transport because the columns in their experiment were saturated from the bottom. In this study, the solubility of Cu, Cd, Pb, Zn, and Ni in all soil layers decreased markedly during the first flooding period, which might be due to Cu, Cd, Pb, Zn, and Ni precipitation in the form of poorly soluble sulfide minerals after SO_4_^2−^ had been reduced to S^2−^ (Weber et al. 2009; de Livera et al. 2011; Fulda et al. 2013). With x ranging between 1.22 and 1.29 for Cu_x_S mineral stoichiometry (Pattrick et al. 1997; Pan et al. 2016) and a stoichiometry of 1:1 for Cd:S, Pb:S, Zn:S, and Ni:S minerals (Allison et al. 1991), there would be enough sulfur in this paddy soil to fully precipitate the reactive forms of all five trace metals as sulfide minerals when all available S–SO_4_^2−^ would be reduced to S^2−^ (Table 1). Additionally, the increase in pH during flooding (Fig. 3) results in an increased trace metal binding to SOM, contributing to the observed decrease in the trace metal solubility. This might especially be relevant for the water layer and the interface between the soil surface and the water layer where no sulfate-reducing conditions were observed (Fig. 3).

During the first drainage period of our paddy soil, the trace metal concentrations increased in all soil layers, now probably due to the oxidation of sulfide minerals after re-aeration of the soil and the development of aerobic conditions and the decrease in pH (Fig. 3). During the second flooding period, the trace metal concentrations in all soil layers decreased again. For the interface between the soil surface and the water layer and the soil layer at a depth of 5 cm, this is the result of an increase in pH, because the Eh values were too high to expect sulfate-reducing conditions (Fig. 3) and consequent precipitation of trace metal sulfide minerals. For the two lower soil layers, however, precipitation of trace metal sulfide minerals might just be possible according to the measured Eh values in these layers (Fig. 3). The Ni concentrations in the bottom soil layer are remarkably high. Since Ni is the trace metal which is the least likely to form sulfide minerals among the five trace metals considered here (Weber et al. 2009; Pan et al. 2016), Ni solubility in this paddy soil might not have been controlled by Ni sulfide minerals but rather by binding of Ni to organic matter in the form of DOM and SOM. The elevated DOC concentrations in the bottom soil layer (Fig. 3) are probably the cause of the higher Ni concentrations (Pan et al. 2016). During the second drainage period, the solubility of the trace metals increased again in all soil layers, probably because of re-aeration of the potted soil and the decrease in pH (Fig. 3).

### Uptake of trace metals by different rice plant tissues during the major growth stages

**Fig. 5:**
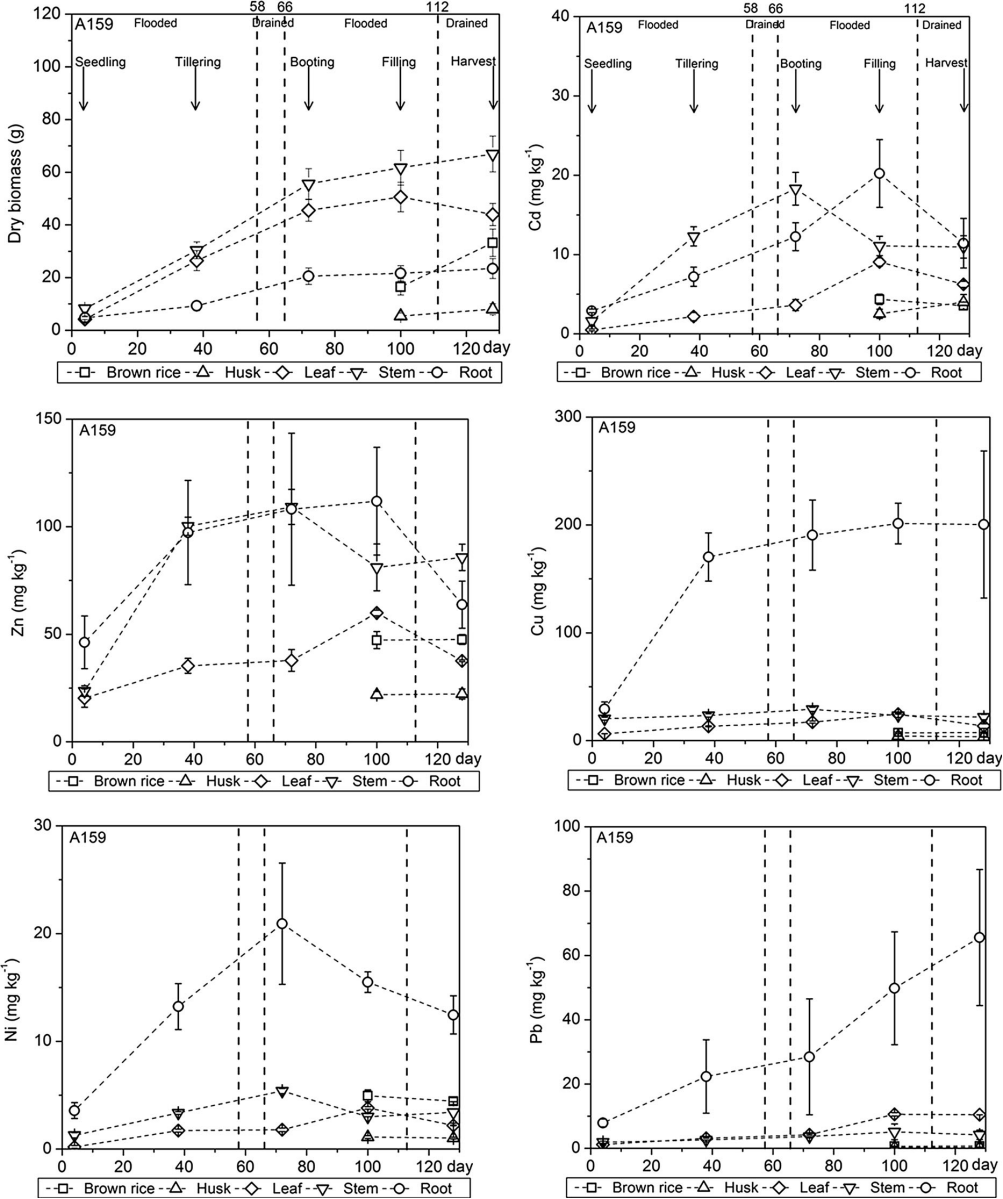
Biomass of the roots and the above-ground plant tissues including brown rice, husks, leaves, and stem and trace metal contents in these plant tissues for rice cultivar A159 during the major rice growth stages. Husks and brown rice were only harvested at the filling stage and final harvest. *The numbers at the top* of the figure indicate the time at which the drainage and flooding periods started

In Fig. 5, the biomass of the different rice plant tissues (roots, stem, leaves, husks, and brown rice) and the trace metal contents in these tissues are presented for the pot experiment with rice plant cultivar A159. The same data for cultivar A16 are presented in the Appendix (Fig. 9). The biomass of the roots and the above-ground plant tissues (stem and leaves) increased at a constant rate but the rate of increase leveled off (root and stem) or the biomass even decreased (leaves) after the booting stage in the second flooding period. For the filling stage and final harvest, the husks and brown rice were included in the sampling of the above-ground plant tissues and these tissues contributed about 50 % to the total above-ground biomass.

The Cd content in the different plant tissues increased at a constant rate but decreased after reaching a maximum at the booting or filling stage in the second flooding period. For the Zn content in the leaves, a similar trend can be observed. The increase in the Zn content in the stem and roots, however, started to lessen after the tillering stage in the first flooding period which was followed by a decrease after the booting or filling stage. The Cd and Zn contents in the husks and brown rice remained constant during the filling stage and final harvest. The increase in the Cu, Ni, and Pb contents in most plant tissues was constant during the first flooding and drainage cycle. For the roots, however, the increase in the Cu content started to level off after the tillering stage, whereas the increase in the Pb content in the roots was constant throughout the pot experiment. The Cu and Ni contents in most plant tissues decreased after the booting or filling stage. Interestingly, the Cu, Ni, and Pb contents in the roots increased at a much higher rate than those in the stem and leaves during the first flooding and drainage cycle. For Cd and Zn, this difference between trace uptake by the roots on the one hand and trace metal accumulation in the stem and leaves on the other was either much less pronounced or even absent. Indeed, large differences are found when calculating the ratios of trace metals in the stem, leaves, husks, and brown rice to trace metals in the roots for Cd, Zn, Cu, Ni, and Pb. For the final harvest, the ratios of Cd and Zn are much higher than the ratios of Cu and Pb for both cultivars (Tables 2, 3). These results reflect a higher translocation of Cd and Zn from the roots into the above-ground plant tissues than for Cu and Pb. In most cases, the ratios for Ni are higher than for Cu and Pb but lower than for Cd and Zn, meaning that Ni has an intermediate translocation behavior. Hence, root uptake and translocation of Cu and Pb into the above-ground plant tissues were strongly regulated, whereas Cd and Zn translocation was more efficient. However, we measured total trace metal contents in the roots in our study and did not distinguish between trace metals adsorbed to the outer root surface and trace metals internalized within the roots, as done before by Kalis et al. (2007) and Duffner et al. (2013) for grass and wheat, respectively. Consequently, it is not possible to be conclusive on whether the exclusion of Cu and Pb from the above-ground plant biomass was due to a mechanism regulating root uptake or an internal mechanism controlling the transfer of Cu and Pb internalized within roots into the above-ground tissues. The Cu, Ni, and Pb contents in the husks and brown rice were constant during the filling stage and harvest. Total Cd uptake by cultivar A159 at the final harvest represents 4.6 % of the total Cd pool in our paddy soil. This is higher than for the other trace metals: 1.6 % for Zn, 0.3 % for Cu, 0.4 % for Ni, and 1.1 % for Pb. Similar results were obtained for cultivar A16. Since the potential of this cultivar to accumulate Cd is lower than for cultivar A159 (Hu et al. 2013), the difference between the relative Cd uptake and the uptake of the other trace metals was less prominent (results not shown) than for cultivar A159. These results agree with the size of the reactive trace metal pools relative to the total trace metal pools in our paddy soil. In this soil, Cd was largely present in reactive forms, whereas the relative size of the reactive Zn, Cu, Ni, and Pb pools was much lower (see “Physico-chemical soil properties” section).

**Table 2:** Ratios of the trace metal contents in the above-ground rice plant tissues versus those in the roots for rice plant cultivar A159 at the final harvest (day 128)

Ratios	Cd	Zn	Cu	Ni	Pb
Stem:root	0.96 ± 0.15	1.34 ± 0.15	0.11 ± 0.02	0.27 ± 0.04	0.06 ± 0.02
Leaf:root	1.09 ± 0.09	1.18 ± 0.08	0.13 ± 0.02	0.35 ± 0.02	0.32 ± 0.04
Husk:root	0.35 ± 0.07	0.35 ± 0.07	0.02 ± 0.004	0.08 ± 0.02	0.005 ± 0.001
Grain:root	0.31 ± 0.05	0.75 ± 0.06	0.04 ± 0.001	0.36 ± 0.007	0.01 ± 0.002

**Table 3:** Ratios of the trace metal contents in the above-ground rice plant tissues versus those in the roots for rice plant cultivar A16 at the final harvest (day 128)

Ratios	Cd	Zn	Cu	Ni	Pb
Stem:root	0.99 ± 0.26	1.28 ± 0.15	0.12 ± 0.04	0.16 ± 0.04	0.03 ± 0.01
Leaf:root	0.72 ± 0.10	1.17 ± 0.12	0.17 ± 0.02	0.46 ± 0.11	0.19 ± 0.08
Husk:root	0.20 ± 0.04	0.33 ± 0.07	0.03 ± 0.01	0.08 ± 0.02	0.01 ± 0.002
Grain:root	0.27 ± 0.06	0.52 ± 0.09	0.05 ± 0.01	0.24 ± 0.08	0.003 ± 0.001

### Cadmium accumulation in rice grains and food quality standards

The Cd content in the rice grains of rice plant cultivar A159 at the final harvest was 3.6 mg kg^−1^ and exceeds about 19 and nine times the food quality standards (FQS) set by the P.R. China and World Health Organization (WHO) (i.e., 0.2 mg Cd kg^−1^) and Japan and Taiwan (i.e., 0.4 mg Cd kg^−1^), respectively (Fig. 5). Although cultivar A16 is known to have a lower potential to accumulate Cd in rice grains than cultivar A159 (Hu et al. 2013), the Cd content reached 2.5 mg kg^−1^ and still exceeds about 13 times the FQS from the P.R. China and WHO and six times the FQS from the Japan and Taiwan (Fig. 9). In our study, the rice grains were not polished before chemical analysis and as a consequence, the Cd content was determined here in brown rice, whereas most people eat polished white rice. However, this is not expected to affect our results, because Masironi et al. (1977) and Kokot and Phuong (1999) found very similar Cd contents in unpolished and polished rice grains. So despite the low solubility of Cd in soil solution during both flooding periods (Fig. 4), still significant uptake and accumulation of Cd in the rice grains of both cultivars occurred to levels far above the FQS. Therefore, human exposure to Cd resulting from the consumption of rice cropped on this contaminated paddy soil might be unavoidable, even when using cultivar A16 which exhibits a lower potential to accumulate Cd than cultivar A159 (Hu et al. 2013). Both cultivars tested here are Indica varieties which are commonly grown in Zhejiang Province, P.R. China (Hu et al. 2013). However, Indica varieties are known to have a substantially higher potential to accumulate Cd in rice grains than Japonica varieties (Römkens et al. 2009b). Hence, it remains unclear whether human exposure to Cd might be reduced when cropping Japonica varieties on our paddy soil.

### Water management effects on trace metal uptake by rice plants

**Fig. 6:**
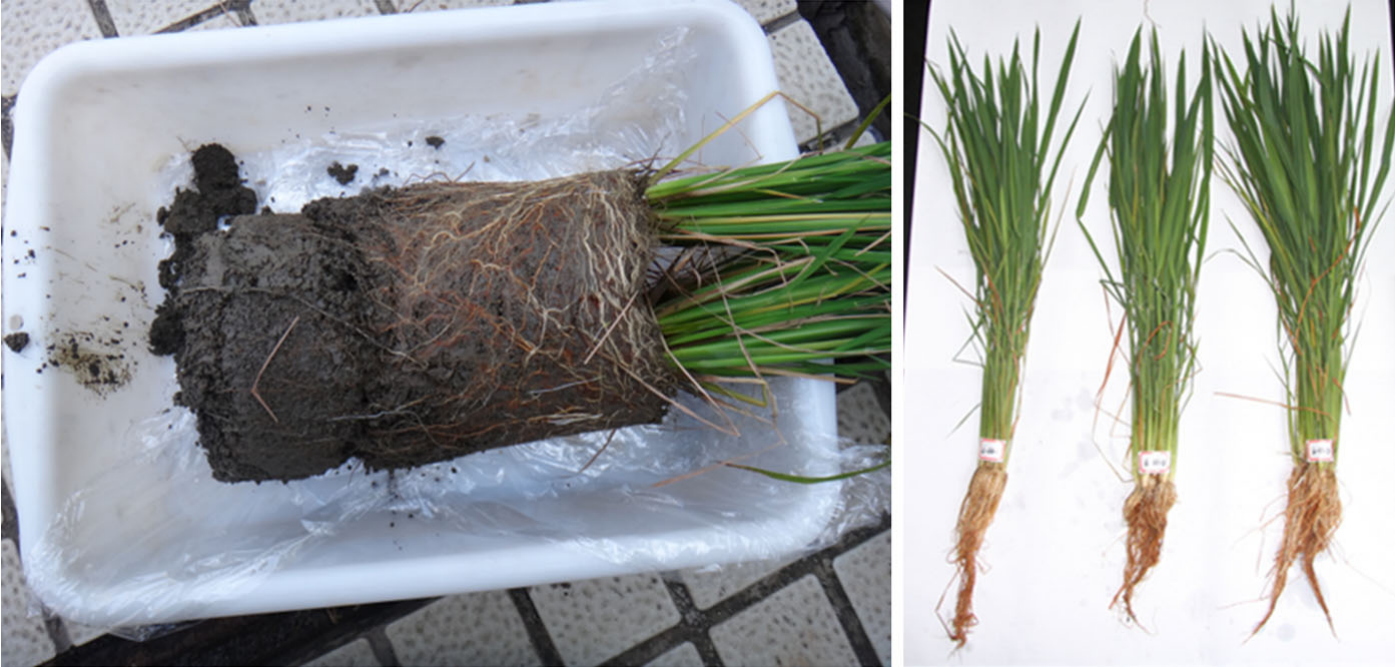
Photographs taken from the rice plants harvested at the rice tillering stage (day 38) showing iron plaque formation on the root surface of rice plant cultivar A159

**Fig. 7:**
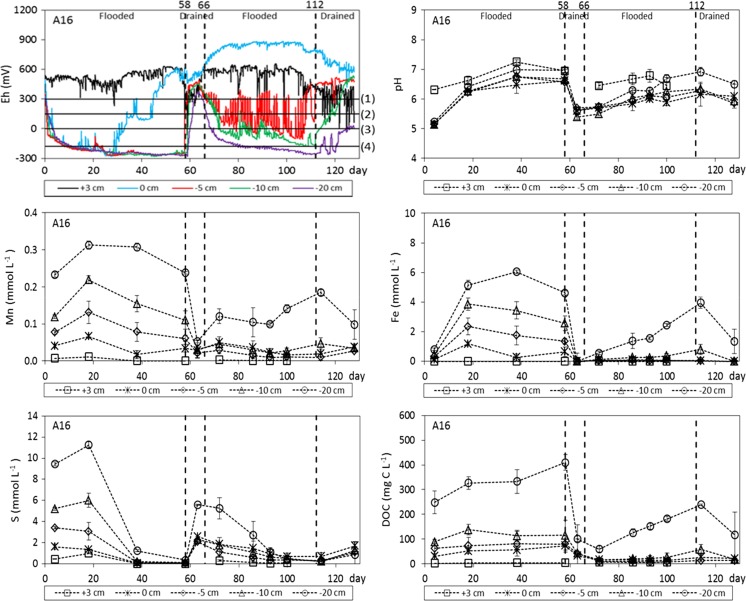
Eh, pH, and Mn, Fe, S, and DOC concentrations in the soil solution for the pot experiment with rice plant cultivar A16 (+3 cm refers to 3 cm above the interface, 0 cm equals the interface between the soil surface and the water layer, and −5, −10 cm, −20 cm refer to 5, 10, and 20 cm below the interface, respectively). *The numbers at the top* *x*-axis of *the two upper figures* indicate the time at which the drainage and flooding periods started. In figure with Eh data, 1–4 refer to the following reactions: (1) denitrification of NO_3_^−^ to N_2_, (2) and (3) reductive dissolution of MnO_2_ and Fe(OH)_3_ to Mn^2+^ and Fe^2+^, respectively, and (4) reduction of SO_4_^2−^ to S^2−^

**Fig. 8:**
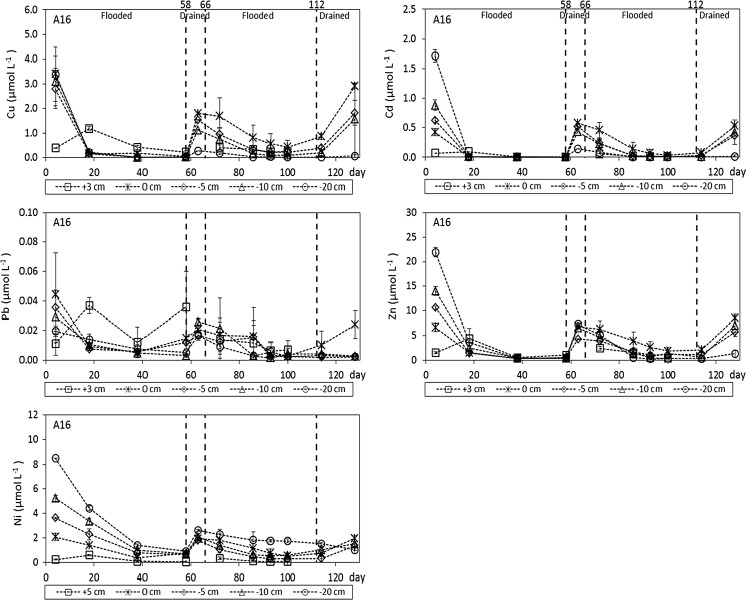
Total dissolved concentrations of Cu, Cd, Pb, Zn, and Ni in the soil solution for the pot experiment with rice plant cultivar A16 (+3 cm refers to 3 cm above the interface, 0 cm equals the interface between the soil surface and the water layer, and −5, −10 cm, −20 cm refer to 5, 10, and 20 cm below the interface, respectively). *The numbers at the top x*-axis of *the two upper figures* indicate the time at which the drainage and flooding periods started

**Fig. 9:**
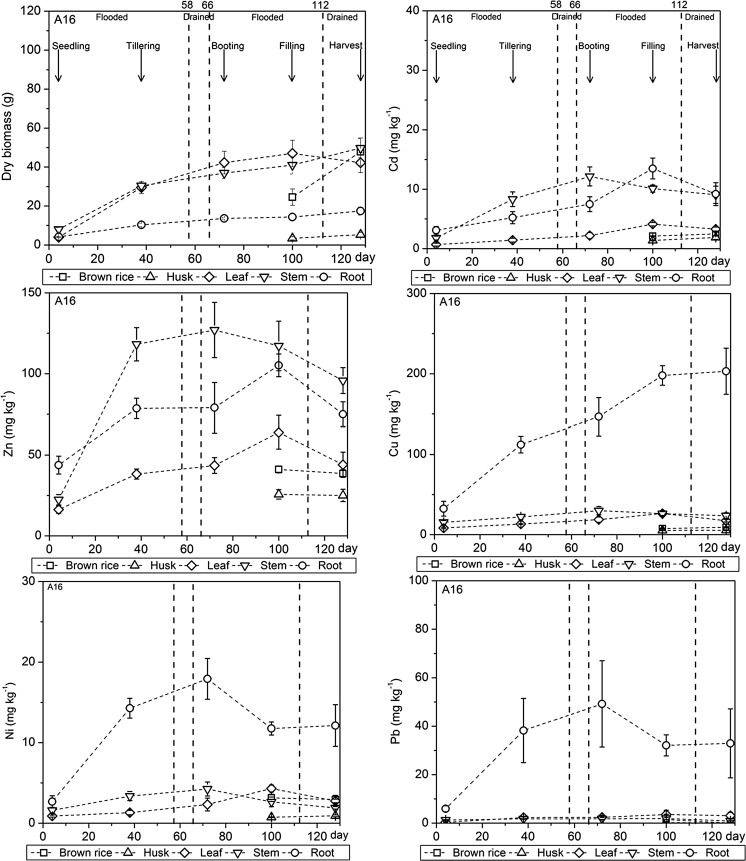
Biomass of the roots and the above-ground plant tissues including brown rice, husks, leaves, and stem and trace metal contents in these plant tissues for rice cultivar A16 during the major rice growth stages. Husks and brown rice were only harvested at the filling stage and final harvest. *The numbers at the top* of the figure indicate the time at which the drainage and flooding periods started

We expected to find a clear impact of water management of the paddy soil in the pot experiment on the uptake of trace metals by the rice plants over time, with trace metal contents in the various rice plant tissues increasing during both drainage periods when the solubility of trace metals showed a large increase (Fig. 4), as has been found in previous studies (Kashem and Singh 2001; Simmons et al. 2008). Instead, trace metal contents in the roots and above-ground plant tissues seemed to increase at a reasonably constant rate during the first flooding and drainage cycle and decreased after reaching a maximum value at later growth stages in many cases. As such, our experimental results did not show a relation between the high temporal variability in the solubility of trace metals and their uptake by rice plants over time. In our pot experiment, total dissolved trace metal concentrations were measured in soil solution sampled by SMS, which were installed within the bulk soil, whereas plant uptake of trace metals is known to occur predominantly from the rhizosphere (Shuman and Wang 1997). During the second flooding period, the Eh at a depth of 5 cm below the soil surface had a high temporal variability (Fig. 3), which can be explained by leakage of O_2_ from the rice plant roots. This O_2_ leakage apparently maintained aerobic conditions in the rhizosphere, because precipitation of Fe^3+^ in the form of Fe-(hydr)oxides was observed as a thin red layer on the root surface of both cultivars harvested from the pot experiment. This phenomenon is called iron plaque formation and is ubiquitous for wetland plants such as rice (Weis and Weis 2004; Liu et al. 2006). Iron plaque formation was not limited to the second flooding period when a diurnal pattern in Eh at a depth of 5 cm below the soil surface was measured in the bulk soil but occurred during the first flooding period as well when such a pattern was not detected and apparently limited to the environment close to the roots (Fig. 6). The presence of iron plaque extended over the entire root length of about 5–15 cm for both cultivars. Although the role of iron plaque itself on root uptake and translocation of Cd into shoots of rice plants has been suggested to be insignificant (Liu et al. 2007), the presence of iron plaque on the root surface of both rice plant cultivars in our pot experiments can only be explained from aerobic conditions prevailing in the proximity of the roots. The solubility of trace metals in this local aerobic soil environment surrounding the rice plant roots is likely to be higher than in the anaerobic bulk soil under flooded conditions when trace metal solubility was controlled by sulfide minerals. The aerobic conditions maintained near the roots might have facilitated a continuous supply of trace metals to the root surface, causing the uptake of trace metals by rice plants to be constant in time. Consequently, trace metal solubility in the rhizosphere should be taken into account to further the understanding of water management effects of paddy fields on the uptake of trace metals by rice plants.

## Conclusions

Two successive flooding and drainage cycles of a field-contaminated paddy soil greatly affected the solubility of trace metals at different depths along the soil profile. During flooding, the solubility of Cd, Zn, Cu, Ni, and Pb in all soil layers decreased markedly, due to the precipitation of trace metal sulfide minerals under anaerobic conditions. Despite its low solubility during flooding, the Cd content in the rice grains exceeds for both cultivars by far the FQS set by individual countries and the WHO. The increase in the trace metal contents in the roots, stem, and leaves was reasonably constant during the first flooding and drainage cycle, but the trace metal contents decreased after reaching a maximum value during the second cycle in most cases. As such, the high temporal variability in trace metal solubility was not reflected in trace metal uptake by rice plants over time. This is probably caused by the presence of aerobic conditions near the root surface, leading to a higher trace metal solubility in the rhizosphere than in the bulk soil and a continuous supply of trace metals to the root surface over time. Consequently, trace metal solubility in the rhizosphere should be taken into account when quantifying the effects of water management on the uptake of trace metals by rice plants. To enable rice production on trace metal contaminated paddy soils, either selection and breeding of rice cultivars further minimizing Cd accumulation or in situ immobilization of trace metals by using stabilizing amendments should be investigated as a measure to mitigate human health risks from trace metal contamination.

## Appendix

See Table 3 and Figs. 7, 8 and 9.
